# Age-specific MRI brain and head templates for healthy adults from 20 through 89 years of age

**DOI:** 10.3389/fnagi.2015.00044

**Published:** 2015-04-08

**Authors:** Paul T. Fillmore, Michelle C. Phillips-Meek, John E. Richards

**Affiliations:** ^1^Department of Communication Sciences and Disorders, University of South CarolinaColumbia, SC, USA; ^2^Department of Psychology, University of South CarolinaColumbia, SC, USA; ^3^Department of Psychology, Limestone CollegeGaffney, SC, USA; ^4^Department of Psychology and Institute for Mind and Brain, University of South CarolinaColumbia, SC, USA

**Keywords:** MRI, aging, adult, segmentation, templates

## Abstract

This study created and tested a database of adult, age-specific MRI brain and head templates. The participants included healthy adults from 20 through 89 years of age. The templates were done in five-year, 10-year, and multi-year intervals from 20 through 89 years, and consist of average T1W for the head and brain, and segmenting priors for gray matter (GM), white matter (WM), and cerebrospinal fluid (CSF). It was found that age-appropriate templates provided less biased tissue classification estimates than age-inappropriate reference data and reference data based on young adult templates. This database is available for use by other investigators and clinicians for their MRI studies, as well as other types of neuroimaging and electrophysiological research.^1^

## Introduction

Morphological and volumetric brain changes that occur with aging are well-documented (see Galluzzi et al., 2008 for a recent review). Despite these changes, many studies aimed at identifying volumetric changes use reference data for tissue segmentation solely derived from young adults. The issues associated with using young adult reference data have been documented and studies have shown that study-specific reference data can improve segmentation results (Ashburner and Friston, 2000; Good et al., 2001; Thompson et al., 2001; Huang et al., 2010). In the current study, we suggest the use of age-specific reference data as an alternative to young adult or study-specific templates. We created and tested a database of age-specific reference templates. Findings suggest that age-specific reference templates can be used to increase segmentation accuracy in developmental neuroscience, neuropsychology and neurology research and clinical practice.

Volumetric brain changes occur with change in age during adulthood. Fotenos et al. (2005) measured whole brain volume differences from age 30 and reported a mean decline in total volume of −0.45% per year after age 65. A common strategy is to distinguish the changes in partial volume estimates (PVEs) of gray matter (GM), white matter (WM), and less often, cerebrospinal fluid (CSF). The most consistent partial volume change is a reduction in GM volume. Ge et al. (2002) reported % GM decline in subjects beginning at age 20 with a constant linear reduction across the span of early to late adulthood. Good et al. (2001), Sato et al. (2003), Sullivan et al. (2004), Taki et al. (2004), Smith et al. (2007) and Lemaître et al. (2005) reported similar negative linear relationships between cortical GM volume and age. Most of these studies show decline in GM beginning about age 20. However, recently Richards and Xie (2015) found that the decline in GM actually begins in the early adolescent years. Changes in WM volume have been found but are not as consistent in the literature. Ge et al. reported % WM changes in a quadratic pattern, with slight increases until age 40, and decreases thereafter. Salat et al. (2009) also reported a quadratic relationship between WM volume and age, with relative preservation or rise in volume until the late 50 s, followed by a steep decline. Lemaitre et al. reported significant WM decline (1.7 cm^3^/year) in their subjects aged 63–75 years. In contrast, Good et al., Sato et al., Sullivan et al., and Taki et al. reported no global WM loss in their subjects. An associated linear increase in CSF volume has also been reported. This finding is consistent across volumetric studies that report CSF results (Good et al., 2001; Lemaître et al., 2005; Smith et al., 2007).

Tissue segmentation is a crucial step for these volumetric studies. These studies classify brain tissue into three primary types: GM, WM, and CSF (or “other matter”, OM). Typically these studies used automated or semi-automated procedures for guiding the segmentation (e.g., FSL’s FAST, Zhang et al., 2001; SPM’s USN, Ashburner and Friston, 2005; Freesurfer, Dale et al., 1999; Atropos, Avants et al., 2011b). These automated procedures usually require *a priori* reference data (Zijdenbos et al., 2002) to guide tissue classification. These spatial priors generally come from a common anatomical reference template. This requires that individual structural brain images be first registered to the common anatomical space, a process known as spatial transformation or normalization. Accurate spatial normalization to a common template confers many advantages, in that it facilitates inter-subject, inter-sample and inter-population comparisons (Evans, 2005). Thus, an important piece in the accuracy of this process is the choice of both templates and normalization methods which can provide a good match between the population of interest and the participants under study. The most often-used MRI template was created by the Montreal Neurological Institute (MNI; Evans and Collins, 1993; Mazziotta et al., 2001a,b). The original MNI template was developed from 305 MRIs, with another one developed later from 152 MRIs. Each used MR images of participants with an average age of about 23 years. Some of the volumetric studies mentioned (Sato et al., 2003; Taki et al., 2004; Mandal et al., 2012) relied on this reference data for their analyses.

Using a template derived from the brains of young adults for analyses with the brains of older adults can be problematic. As Huang et al. (2010) point out, despite the widespread use of the MNI and other such normalizing templates, inter-subject variation in brain structures for special populations such as older adults can result in less than ideal compensation for brain region discrepancies after spatial normalization with these templates. An alternative to using the MNI template is to create a study-specific template, derived from structural images of the participants in the study. To demonstrate this, Huang et al. used a study-specific template rather than one developed from young adult images for spatial normalization within a functional MRI (fMRI) data analysis. Huang et al. found that more voxels were identified as significant in older adults when a study-specific template was used. The main advantage conferred by the construction of study-specific templates in this case is a very precise mapping to the common space, since it is derived from the actual participants of the study.

Study-specific templates have also been implemented within voxel-based morphometry (VBM; e.g., Peelle et al., 2012). VBM is an automated method used for voxel-wise statistical investigation of focal differences in brain anatomy as measured by MRI (Ashburner and Friston, 2000). In this context, creating a study-specific template may involve segmenting a group of images from the sample with MNI tissue priors, averaging those partial volumes to come up with study-specific tissue priors, and then using those priors to re-segment all of the images in the sample (see Lemaître et al., 2005 for an example). Studies comparing a study-specific template to the MNI template for VBM demonstrated that using a study-specific template reduced anatomical biases in the analysis (Ashburner and Friston, 2000; Good et al., 2001; Thompson et al., 2001). Again, it is likely that this is due to the precision in mapping afforded by a study-specific template. Recent work has shown that the use of high-resolution nonlinear spatial normalization tools further reduces these biases (Callaert et al., 2014).

A study-specific template is an improvement over the MNI template, but a second, possibly better, solution is the development of age-appropriate *a priori* reference data. This solution confers several advantages over study-specific templates. First, age-appropriate templates can be used over multiple studies allowing for easier study-to-study comparisons. Results using study-specific templates cannot easily be compared to the results of another study using a different study-specific template. Age-appropriate templates alleviate this issue. Second, a study-specific template may be created by averaging images from a broad age range, depending on the study goal. Age-appropriate templates can be created in smaller increments and may be more similar to any one individual image than a broad study-specific template (c.f. Rohlfing et al., 2009). Several age-appropriate templates have been done for pediatric ages (Wilke et al., 2003; Altaye et al., 2008; Yoon et al., 2009; Fonov et al., 2011; Sanchez et al., 2012a). Age-appropriate templates have been used with pediatric neuroimaging data and improve classification of tissue types in pediatric samples (Wilke et al., 2003; Sanchez et al., 2012a,b).

There are a few reported age-specific templates for adults. Lemaître et al. (2005), created a T1-weighted probabilistic brain atlas representing an average of 662 subjects aged 63–75 years. These subjects were a sub-sample of the Epidemiology of Vascular Aging cohort, a longitudinal study on vascular aging and cognitive function in healthy older adults (see Dufouil et al., 2001 for details of study). MR images for these subjects were acquired between 1995 and 1997 on a 1.0T MR scanner. Farrell et al. (2009) created four age-specific templates, T1- and T2-weighted templates representing 54 subjects aged 65–70 years and 25 subjects aged 75–80 years. These subjects were sub-samples of the “National Aging Brain” Study cohort (MacLullich et al., 2002) and the “Simpson’s Study” cohort (Shenkin et al., 2003), both studies of normal aging. The “National Aging Brain” subsample MRIs were collected on a 1.9T scanner. Sato et al. (2003) created reference templates for a very wide age range that included older adults: 10 age-specific templates created from 1,600 Japanese subjects aged 20–80 years. Reference templates were created with the T1-weighted images, acquired on a 0.5T MR scanner. More recently, Rorden et al. (2012) created matching CT and MRI templates for use with stroke-aged populations. Fifty MRI scans were used (mean age of participants 72.9 years) all acquired on a 3T scanner. Perhaps most relevant to the current work, Rohlfing et al. (2009) also proposed the use of age-specific, as well as sex-specific, templates. Based on 64 subjects (30 M/34 F) across various ages, they used a combination of regression models and non-rigid registration methods to show that decade-based age-specific templates provided increased accuracy when compared with a study-wide template. The current study follows the strategy of these studies to create age-appropriate templates. The current investigation constructed age-specific brain templates for adults 20–89 years of age. The procedure for template construction involved gathering images from open sources of adult MRIs and using a state-of-the-art non-linear iterative averaging method. The MR images were compiled from our own acquired MRI images, the Pediatric MRI Data Repository created by the NIH MRI Study of Normal Brain Development (NIHPD; Evans and BDCG, 2006; Almli et al., 2007; Waber et al., 2007), the Information Extracted from Medical Images (IXI) database (Heckemann et al., 2003; Ericsson et al., 2008), and the Open Access Series of Imaging Studies (OASIS; Marcus et al., 2007, 2010) cross-sectional and longitudinal image sets. We compared the use of age-specific templates to young adult and age-inappropriate templates for brain tissue segmentation, with the hypothesis that age-specific templates will provide less biased tissue segmentation. We did this by using the manually segmented images from the Internet Brain Segmentation Repository (IBSR; Filipek et al., 1994; Rohlfing, 2012). Manual segmentation is often considered a “gold standard” for tissue segmentation, though this process is both time-consuming and subjective (Dale et al., 1999). However, results from automated procedures may be compared with the manual segmentation to compare the relative accuracy of the automated procedures (e.g., Atkins et al., 2002; Valverde et al., 2015). We also demonstrated the potential usefulness of the age-specific templates by highlighting their ability to confirm characteristics of brain aging. We have previously constructed age-specific templates for 2 weeks through 4 years of age (Sanchez et al., 2012a) and 4.5 through 20–24 years of age (Sanchez et al., 2012b). Thus together with the current study, we have constructed a database of normative age-appropriate average MRI templates across the lifespan (Richards and Xie, 2015). These templates (head, brain, and segmented priors) are publicly available through our website.^2^

## Materials and Methods

### Participants

The MRI images came from adults, ranging from 20–89 years of age. The images came from several sources. USC-MCBI: Some participants came from studies performed at the University of South Carolina McCausland Center for Brain Imaging. The USC-MCBI participants (*N* = 132; 50 M/80 F/2 Unknown; 20–65 years were all normal, healthy adults with no history of neurological or psychiatric illness, head trauma with loss of consciousness, or current or past use of psycho-stimulant medications, cardiovascular disease, and no abnormal findings on the MRIs. NIHPD: One set of participants came from the Pediatric MRI Data Repository created by the NIHPD (Evans and BDCG, 2006; Almli et al., 2007; Waber et al., 2007). The NIHPD participants (*N* = 26; 14 M/12 F, 20–24 years) were recruited across six Pediatric Centers using community-based sampling techniques to reflect the gender, income, and race/ethnicity variation in the United States Census 2000. Participants were screened for the presence of behavioral/emotional/academic problems, factors that adversely impact healthy brain development and factors that would prohibit the full completion of the study protocol. IXI: A third set of participants came from the IXI database (Heckemann et al., 2003; Ericsson et al., 2008). These participants (*N* = 546; 241 M/304 F/1 Unknown; 20–86 years) consists of 600 MRIs from normal, healthy adults, with no cognitive impairment, collected at three different hospitals in the London, UK area; cardiovascular or other health status is unknown. OASIS-CS: The fourth set of participant came from the cross-sectional database of the OASIS (Marcus et al., 2007). The OASIS-CS participants (*N* = 283; 102 M/181 F; 20–89 years) came from a study that included “non-demented”, and early stage Alzheimer’s disease. Only those participants who were non-demented were included, and cardiovascular or other health status is unknown. OASIS-LONG: The final set of data came from the longitudinal portion of the OASIS project (Marcus et al., 2010). This data set consists of a longitudinal collection of 150 participants ranging from 60–96 years of age all acquired on the same scanner using identical scan sequences. Each participant was scanned on two or more visits, separated by at least 1 year for a total of 373 imaging sessions. Again, only those classified as “non-demented” were included in the present project. We used a total of 175 scans from 72 participants (22 M/50 F) from the OASIS-LONG project. Repeated scans from the OASIS longitudinal study were used in the construction of the templates for the appropriate age of the scan; we did not keep track of the repeated scans in any of the analyses. Overall 1162 scans from 1059 participants were used for creation of the templates. All projects had institutional review board approval and informed consent for participants.

The participants were grouped into five-year age groups (i.e., 20–24, 25–29, etc.) through age 89 years, 10-year age groups (e.g., 20–29, 30–39, etc.) or multi-year age groups (25–39, 40–59, 60–89). Average templates for the study were created for the three grouping types (5-, 10-, multi-year; see Table 1 for age group information broken down by five-year group).

**Table 1:** **Demographic information by five-year age group broken down by gender and image source**.

Age range	Total *N*	Gender (# Female)	% Female	IXI *n*	MCBI *n*	NIHPD *n*	Oasis *n* Cross-sectional	Longitudinal
20–24	244	139	57	42	88	26	88	0
25–29	101	62	61	58	15	0	28	0
30–34	79	29	37	52	16	0	11	0
35–39	50	22	44	44	1	0	5	0
40–44	61	37	61	47	4	0	10	0
45–49	65	33	51	41	3	0	21	0
50–54	57	38	67	38	2	0	17	0
55–59	73	45	62	55	2	0	16	0
60–64	83	58	70	61	0	0	11	11
65–69	89	54	61	51	1	0	14	23
70–74	101	70	69	37	0	0	24	40
75–79	61	41	67	12	0	0	9	40
80–84	62	43	69	6	0	0	17	39
85–89	36	22	61	2	0	0	12	22
Total	1162	693	60	546	132	26	283	175

Additionally, we used images from the IBSR^3^ to compare the outputs of our automated segmentation routines to those attained by manual segmentations. These images served as an external test set, and were not used for template creation. The IBSR dataset is composed of two image sets: (1) the IBSR20 (Filipek et al., 1994) which was created based on a semi-automated intensity contour mapping algorithm (Kennedy et al., 1989) and manually edited signal intensity histograms (*N* = 18, 10 M/10 F, 23–38 years for data used; two subjects were excluded from the original 20 due to large bias artifacts); and (2) the IBSR 18 (Rohlfing, 2012), which was hand segmented with a three-class model (GM, WM, CSF), and includes both adult and child images. For the current study, we used only the adult data (*N* = 10, 7 M/3 F, 35–71 years).

### MRI Data Acquisition

The procedures for the MRI sequences differed across the datasets. USC-MCBI: The data at the USC-MCBI were collected on a Siemens Medical Systems 3T Trio. The MRI protocol has been described in detail in Sanchez et al. (2012a). Briefly, a 3D T1-weighted “MPRAGE” RF-spoiled rapid flash scan in the sagittal plane was employed with the following parameters: TR = 2250 ms, TE = 4.52 ms, flip angle = 9°, FoV = 256 mm × 256 mm, matrix size = 1 × 1 × 1 mm^3^ (the sagittal dimension of the T1W ranged from 160–212 slices. NIHPD: The procedures for the NIHPD are described in detail by others (Evans and BDCG, 2006; Waber et al., 2007). Briefly, a 3D T1-weighted spoiled gradient recalled (SPGR) echo sequence was employed with following parameters: TR = 22–25 ms, TE = 10–11 ms, flip angle = 30°, FoV = 256 mm IS × 256 mm AP, matrix size = 256 × 256: 1 × 1 × 1 mm^3^ voxels, 160–180 slices of sagittal orientation. The scans were conducted at different sites with Siemens Medical Systems (Sonata, Magnetom) and GE (Signa Excite) scanners. IXI: The IXI data consisted of multislice spin echo T1 images collected at 3 sites with 1.5 and 3T scanners (FoV = 256 mm × 256 mm, matrix size = 0.9375 × 0.9375 × 1.2 mm^3^). OASIS: The OASIS study implemented a T1-weighted MPRAGE on a 1.5T Vision Scanner (TR = 9.7 ms, TE = 4.0 ms, flip angle = 10°, FoV = 256 mm × 256 mm, matrix size = 1 × 1 × 1 mm^3^). IBSR: T1-weighted images, scanner/scan parameters unspecified, with 128 saggital slices (FoV:256 × 256 mm, variable matrix size: 0.8370/0.9375/1.0 × 1.5 × 0.8370/0.9375/1.0 mm^3^).

### File Preparation

The MR images were prepared for processing in three steps. First, the brains were extracted from the whole-head MRI volume using the brain extraction tools of FSL. An automated bash script using the FSL tools (Smith et al., 2004; Woolrich et al., 2009) completed this task with the following actions: register the head to the MNI-152 head (Collins et al., 1995; Mazziotta et al., 2001a); inverse-transform an MNI-brain-mask to the participant space; use the mask to get a preliminary brain; use betsurf to get a binary skull mask; use the skull mask to delineate a second preliminary brain; use bet2 to extract the brain from the second preliminary brain mask for the final brain (Smith, 2002; Jenkinson et al., 2005). We visually inspected each brain for accuracy, and adjusted the bet2 variables (e.g., fractional intensity threshold, center of gravity, starting sphere size) to get a well-formed brain volume (Jenkinson et al., 2005).

Second, we adjusted the MRI intensity variations found in the datasets (NIHPD, USC-MCBI, OASIS, IXI, MUSC) that stemmed from different machines, different recording sites and slight differences in protocol. First, bias field inhomogeneity was corrected with a N4 bias field correction procedure (Tustison et al., 2010; Avants et al., 2011b). Second, the MRI voxel levels were normed so that GM peak intensity was 100. This was done by first segmenting the brain with the FSL FAST procedure, “FMRIB’ s Automated Segmentation Tool” (Zhang et al., 2001) into GM, WM, and OM. The MRI voxels with PVEs of 1.0 in the GM segments were averaged to determine the average voxel intensity for GM. The scan was then renormed with this value to have a value of 100, which resulted in the peak of the GM intensity in the MRI histogram curve equaling 100. These procedures ensured standardization among all the scans (see Bağcı et al., 2010, for a more detailed discussion of intensity standardization).

Third, the individual participant MRI volumes were classified into GM, WM, and OM. We used two initial procedures to do this, in order to compare segmentation used with MNI or no reference: first, the FSL FAST procedure was used to segment the T1W scans without using any prior classification volumes (“Image”) and second, the FSL FAST procedure was used with the MNI priors (“MNI”). Both methods resulted in a separate PVE for GM, WM, and OM, for each participant’s MRI volume.

### Construction of Age-Specific Templates

**Figure 1:**
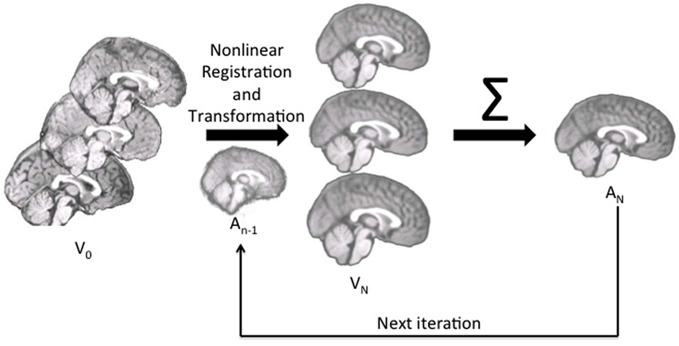
**The pipeline for age-specific template creation.** First, participant brains were rigidly registered to the MNI brain, maintaining the volume and size of the original. Rigidly registered brains (V_0_) were then averaged to create a rough template (A_0_). This template was used as the first registration target, to which each participant brain was nonlinearly registered and transformed (V*_n_*). With each iteration, the participant brains were nonlinearly registered to the new average (A_*n*−1_), transformed and then re-averaged to create a new, relatively more precise average (A*_n_*) for the next iteration.

We constructed the age-specific templates with the iterative routines found in Sanchez et al. (2012a,b); also see Guimond et al., 2000; Yoon et al., 2009; Fonov et al., 2011, for examples of similar iterative routines). The whole-head MRI volumes and brain-extracted MRI volumes were performed separately. Figure 1 is a schematic representation of the steps used in the construction of the template for a specific age group for both whole-head and brain-extracted templates. The first step of the iterative procedure was to construct a tentative average (Figure 1, “A_0_”). A rigid rotation (FLIRT 6 parameter linear registration and transformation; Jenkinson and Smith, 2001) to the MNI-152 adult template ensured all images were oriented in the same way prior to averaging (ICBM-152 defined in Mazziotta et al., 2001a; Joshi et al., 2004). The second step of the iterative procedure consisted of a non-linear registration (ANTS, “Advanced Normalization Tools”; Avants et al., 2008, 2011a) to the current reference average (A_*n*−1_), a transformation of each participant MRI into the template space (V*_n_*), and then a averaging of the transformed MRIs (A*_n_*). This average was then used as the reference model in the next iteration (A_*n*−1_ on next step). The first non-linear registration was done with low resolution (50 × 0 × 0 iterations), the second with medium resolution (50 × 50 × 0 iterations), and the final steps with fine resolution (50 × 50 × 50). The root mean square (RMS) difference between successive average reference models was calculated, and the iterative procedure was done until leveling of the successive RMS values was obtained. The final reference model is the “age-specific” template. More details of this procedure may be found in Sanchez et al. (2012a).

We used open-source and publicly available tools for these methods. An automated image-processing pipeline was constructed with the Linux bash scripting language. The FSL FLIRT tool (Jenkinson and Smith, 2001) performed the rigid rotation to the MNI-152 volume (6-degrees-of-freedom). The ANTS program (Avants et al., 2008, 2011a) performed affine and diffeomorphic registration (symmetric normalization, SyN) of the source volumes to the reference volumes. The ANTS program uses symmetric diffeomorphic normalization to capture shape and intensity differences and enhance alignment of image features. The ANTS tool, AverageImages (non-normed) was used for the averaging step.

The transformation parameters from the averaging process were used to create “Image” and “MNI” average tissue priors. Each iteration step from the ANTS procedure resulted in a coefficient matrix for the affine transformation, three volumes with non-linear deformation values for the forward-transformation (Warp *x*, *y*, *z*) of the source volume to the reference volume, and three MRI volumes with non-linear deformation values for the inverse transformation (InverseWarp *x*, *y*, *z*) of the reference volume to the source volume. The spatial transformation coefficients and deformation-volumes on the final iteration represent the linear and non-linear registration of the individual participant MRI volumes to the average volumes. The individual participant PVE volumes were forward-transformed by the participant-template transformation parameters into the average template size and orientation. An average of the transformed participant volumes was made separately for the GM, WM, and OM volumes for each segmentation procedure in order to create “Image” and “MNI” tissue priors for each age-specific template.

### Tissue Classification Tests

**Figure 2:**
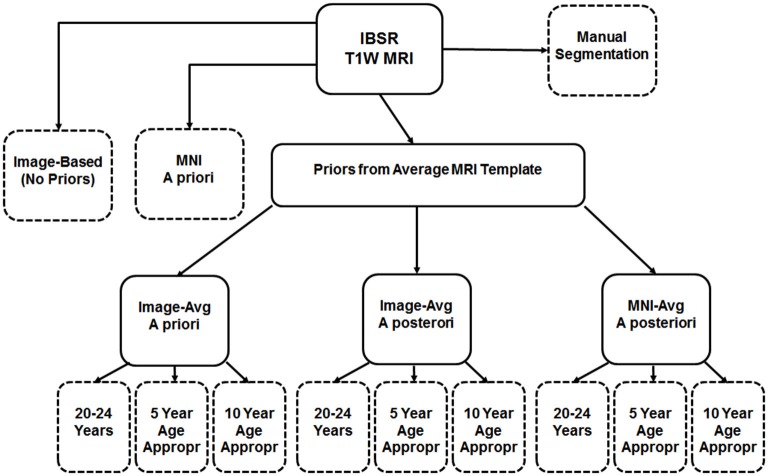
**Schematic of segmentation routines used on the IBSR test subjects**. All subjects had a baseline segmentation with no priors (“Image”), as well as with the MNI152 prior set (“MNI *a priori*”). Using age-based average MRI templates, segmentation with priors specified both *a priori* and *a posteriori* using image-based averages, as well as *a posteriori* using MNI-based averages. Each of these was conducted using priors from three template age groups: a 20–24 year template, a five-year age-matched template, and a 10-year age-matched template. The eleven segmented volumes resulting from this, and the manual segmented volume, are represented with dashed-line outlines.

First we used the manually guided segmentations from the IBSR datasets in order to evaluate the relative fit between our various automated methods and a “gold standard” manually guided segmentation. The goal of this comparison was not primarily an evaluation of the accuracy of these automated methods, but rather to determine the best “proxy standard” to use for our comparisons of age-specific priors, where manual segmentations were unavailable. For this analysis we calculated segmented GM and WM on the IBSR participants MRI volumes for the following eleven procedures: (1) “Image”, calculated from the T1W with no priors; (2) “MNI-*a priori*”: calculated using the MNI template segmented GM/WM as priors only at the beginning step (FAST—A option); (3) “Image-AVG-*a priori*” used the “Image” averaged GM/WM volumes as priors only on the beginning step (FAST–a option); (4) “Image-AVG-*a posteriori*”: used the “Image” averaged GM/WM volumes as priors on the beginning and posteriori steps (“FAST–p”); and (5) “MNI-*a posteriori*”: Used the “MNI-averaged” GM/WM on the beginning and posteriori steps (“FAST–p”). For the analyses using the averaged GM/WM volumes as segmenting priors, we used the priors from the young adult template (20–24 years), the age-appropriate five-year template, or the age-appropriate 10-year template. The Dice coefficient, which measures degree of overlap (ranging from 0, or no overlap, to 1, total overlap; Dice, 1945) and represents the intersection of two similarly labeled regions divided by the mean volume of the regions, was used to compare the outputs of our various segmentations with each manually segmented IBSR volume. Figure 2 is a schematic showing the eleven segmented volumes that were compared against the manually segmented IBSR volume. A list of these volumes is as follows:

1. No priors (Image)
2. MNI priors (MNI-*a priori*)
3. Image-based young adult priors, *a priori* only (Image-AVG-*a priori*)
4. Image-based 5-year age appropriate priors, *a priori* only (Image-AVG-*a priori*)
5. Image-based 10-year age appropriate priors, *a priori* only (Image-AVG-*a priori*)
6. Image-based young adult priors, *a priori* and *a posteriori* (Image-AVG-*a posteriori*)
7. Image-based 5-year age appropriate priors, *a priori* and *a posteriori* (Image-AVG-*a posteriori*)
8. Image-based 10-year age appropriate priors, *a priori* and *a posteriori* (Image-AVG-*a posteriori*)
9. MNI-based young adult priors, *a priori* and *a posteriori* (MNI-AVG-*a posteriori*)
10. MNI-based 5-year age appropriate priors, *a priori* and *a posteriori* (MNI-AVG-*a posteriori*)
11. MNI-based 10-year age appropriate priors, *a priori* and *a posteriori* (MNI-AVG-*a posteriori*)

Second, 56 participant volumes from the original image set were chosen (four selected randomly from each five-year age group, two males and two females) to compare the effect of the age-appropriate and age-inappropriate templates on media segmenting. We constructed segmented PVE’s for each participant using the averaged segmented volumes from five-year templates of successively older and younger five-year age groups. Based on the results from the first analysis, we chose the “Image-AVG-*a posteriori*” method for this calculation (image-averaged priors from age-inappropriate MRI template with priors applied at the beginning step and a posteriorly, FAST–p). The Dice coefficient was used to compare the age-appropriate segmentation with each of the segmentations derived from the younger and older five-year templates.

### Volumetric Analyses

To assess the ability of age-appropriate templates to confirm previously discussed characteristics of brain aging, we calculated GM, WM, and OM volumes (in mm^3^) for all participants using the PVE volumes based on segmentation with age-appropriate five-year template priors. We also calculated GM + WM volume. As a point of comparison, we calculated inner skull volume (also in mm^3^) using results of the betsurf program, a brain extraction tool available in FSL (Smith, 2002). Betsurf produces three additional surfaces: inner and outer skull, and outer scalp. Further information on the betsurf program is available from http://www.fmrib.ox.ac.uk/analysis/research/bet/.

## Results

The database consists of age-specific templates divided into five-year increments, 10-year increments and multi-year age groups (25–39, 40–59, 60–89). Templates for T1W head and T1W brain exist for each age group. Additionally, PVEs and binary-segmented images were created for each template from the two different segmentation methods: Image (no priors) and MNI priors. The T1W average templates represent gray matter values normalized to 100, so that variations in voxel intensity between MRI volumes did not affect the averages. All averaged templates result from an initial linear registration and transformation with the MNI-152 template. Thus, the templates are loosely oriented to the MNI-152 volume. Subsequent steps utilized nonlinear transformations with age-specific templates created from prior steps, which minimized the influence of the MNI-152 template. The resulting age specific head and brain templates represent the average size for the participants at that specific age.

**Figure 3:**
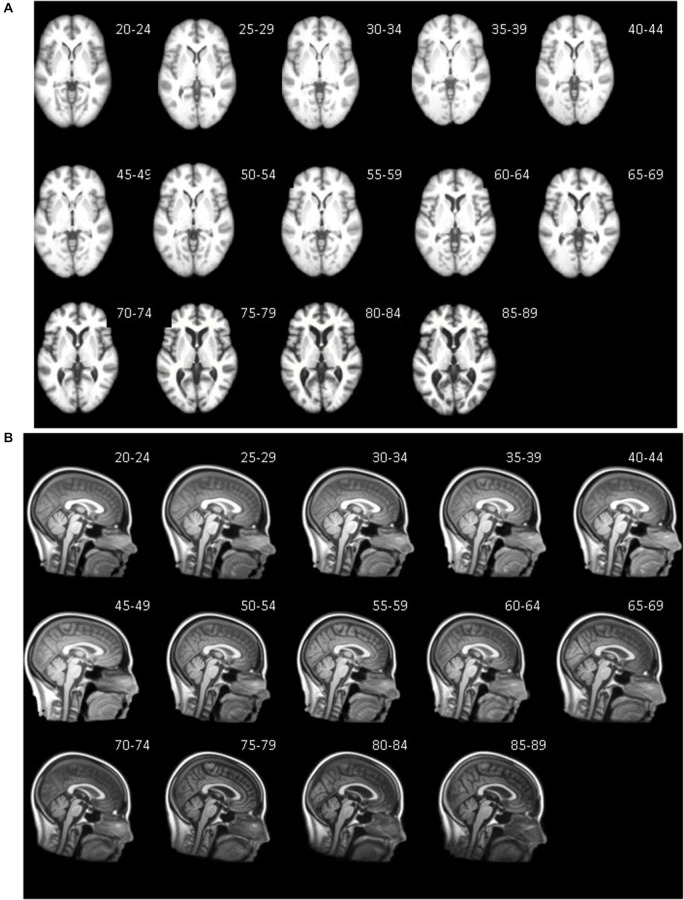
**Age-specific templates showing axial slice at AC-PC commissure (A) of brain extracted average template and mid-sagittal slice (B) of whole head average template**. T1W MRI volumes across ages of study.

Axial (A) and mid-sagittal (B) slice for each five-year age-specific T1W brain (A) and head (B) template are pictured in Figure 3. The sagittal view of the averaged templates exhibits small morphological structures in fine detail, as can be seen with cortical and subcortical anatomy. Gradual brain atrophy is noticeable especially when comparing the templates across age. All templates appear to be relatively consistent in regards to level of detail and clarity.

### Tissue Classification Tests: IBSR and Segmentation Methods

**Figure 4:**
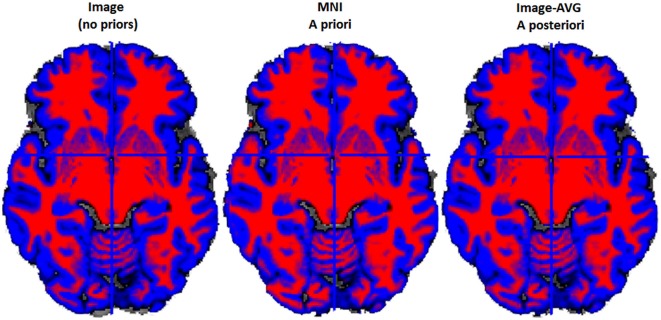
**Example two-class MR volume segmentation from a 60-year-old female**. Volume was segmented and then gray matter (GM) and white matter (WM) were combined into a two-class volume. Red = WM; Blue = GM; Gray = other brain tissue. Label above volume indicates which set of tissue priors was used to segment the volume. Comparing the Image to the MNI, the MNI seems to classify more voxels as WM and fewer voxels as GM. Age-appropriate (“Image-AVG *a posteriori*”) and Image match one another rather closely, however age-appropriate does seem to identify slightly more voxels as GM.

**Figure 5:**
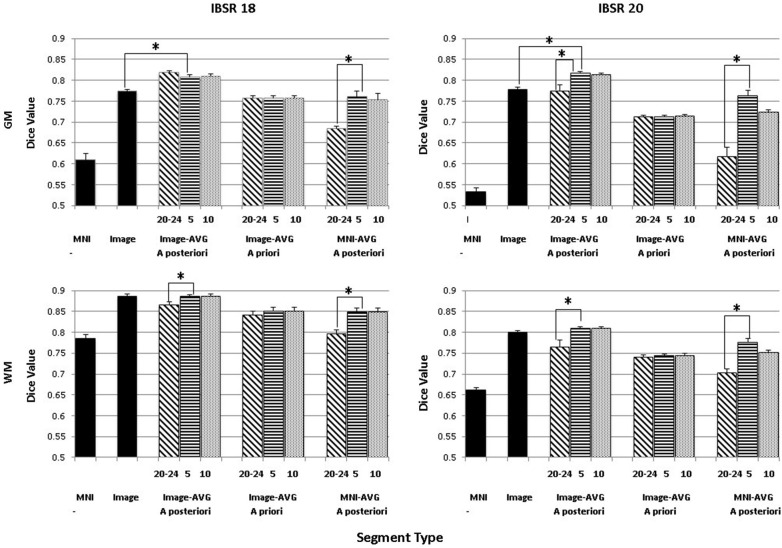
**Comparing segmentation methods across age ranges**. Dice similarity values are shown for both gray matter (GM) and white matter (WM) for comparisons to the manually segmented IBSR18 and IBSR20 datasets. Values are shown for the MNI-based and Image-based prior sets, for *a priori* and *a posteriori* specification, and for age-appropriate five-year (“5”) and ten-year (“10”) templates, as well as our young adult (“20–24”) template. “MNI” refers to the standard MNI152 priors, and “Image” refers to segmentation from the base image, without explicitly specifying priors. Note that performance is generally best for *a posteriori* specification, using age-appropriate priors within a five-year range. Selected significant *post hoc* comparisons are highlighted; the “MNI” template had the lowest Dice values for each comparison.

The purpose of this analysis was to evaluate the overlap between the manually segmented brain volumes from the IBSR and the primary segmentation routines (listed in Figure 2). Figure 4 illustrates the general pattern of the segmentation outputs for a single participant. We used a one-way ANOVA to examine the relative fit of the five methods (Image, MNI-*a priori*, Image-AVG-*a priori*, Image-AVG-*a posteriori*, MNI-AVG-*a posteriori*) to the manually guided segmentations, separately for GM and WM, and for each IBSR dataset. The segment type main effect was significant for all four comparisons; GM, IBSR-18, *F*_(4,41)_ = 42.86, *p* < 0.001; WM, IBSR-18, *F*_(4,45)_ = 16.07, *p* < 0.001; GM, IBSR-20, *F*_(4,34)_ = 139.57, *p* < 0.001; WM, IBSR-20, *F*_(4,34)_ = 81.61, *p* < 0.0001. Figure 5 show the means separately for these five segmentation procedures; and the segmentations with average template priors are shown separately for the young adult, five-year and 10-year age-appropriate average templates. We did *post hoc* tests to compare averages from individual segment types, and there were many significant differences. For GM, segmentation with age-appropriate five-year template was the best fit and the participant’s individual MNI segmentation was the worst fit. A particularly relevant comparison is the Image and the Image-AVG-*a posteriori* comparison, which showed a significantly larger dice value for the age-appropriate segmentation. The same general pattern occurred for WM, with the exception that for WM, segmentation with age-appropriate Image-AVG-*a posteriori* method was not significantly different from the Image (no priors) segmentation (see Figure 5, bottom figures).

A second analysis based on these data was a comparison among the three segmentation methods with that used priors from the average MRI templates (Image-AVG-*a priori*, Image-AVG-*a posteriori*, MNI-AVG-*a posteriori*). A segment type (3) X age-type (3: young adults, five-year age-appropriate, 10-year age-appropriate) ANOVA was done on the Dice value. The segment type main effects are a subset of the prior analysis, and so will not be considered. For both media types and IBSR groups, there were significant main effects of age-appropriate type, GM, IBSR-18, *F*_(2,36)_ = 16.32, *p* < 0.001; WM, IBSR-18, *F*_(2,36)_ = 72.8, *p* < 0.001; GM, IBSR-20, *F*_(2,66)_ = 75.55, *p* < 0.001; WM, IBSR-20, *F*_(2,66)_ = 55.54, *p* < 0.0001. We tested *post hoc* comparisons by examining the three age-appropriate types for the three segmenting types. For the Image-AVG-*a posteriori* method, the Dice value for the five-year and 10-year age appropriate averages were not statistically significant; whereas the Dice value for the two age-appropriate averages were significantly larger than the young adult average for the WM for IBSR-18 and IBSR-20 participants, and for the GM for the IBSR-20 participants (see Figure 5). All four comparisons for the MNI-AVG-*a posteriori* showed this pattern (5 year = 10 year > young adults); there was no significant difference among the age-appropriate types for the Image-AVG-*a priori* segmenting type.

### Tissue Classification Tests: Younger and Older Age Average Priors

**Figure 6:**
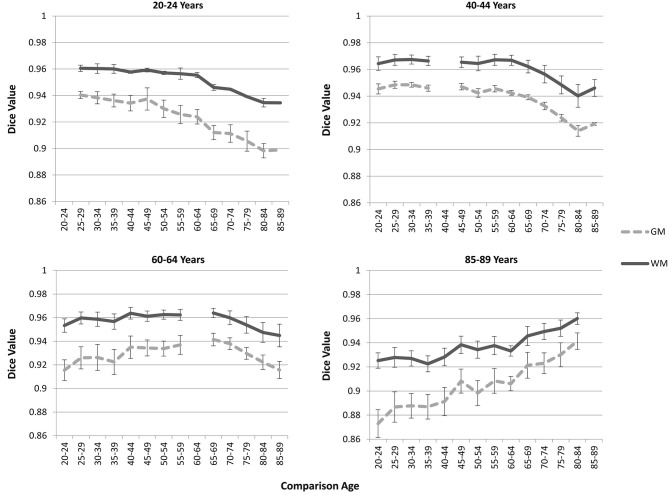
**Age-appropriate segmentation priors compared to those from inappropriate ages**. Partial volume estimates (PVE’s) for both gray matter (GM) and white matter (WM) were derived from segmentation using age appropriate (five-year range) priors, as well as priors derived from younger and older age groups. These PVE’s were compared, and mean Dice similarity values are shown for several representative ages. Note the consistent drop off in similarity, as utilized priors grow farther from the appropriate age range.

**Figure 7:**
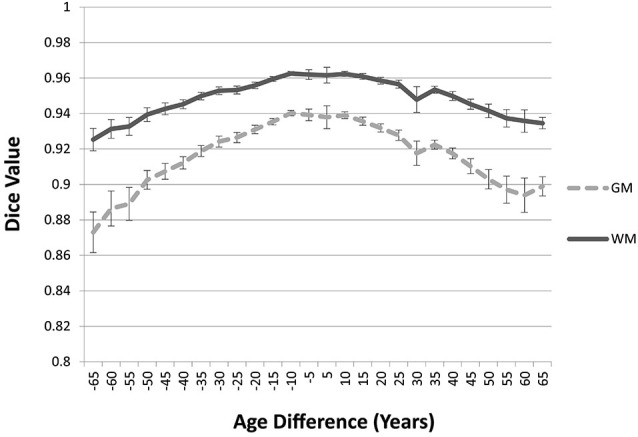
**Age-appropriate segmentation priors compared to those from priors of different ages**. Partial volume estimates (PVE’s) for both gray matter (GM) and white matter (WM) were derived from segmentation using age appropriate (five-year range) priors, as well as priors derived from younger and older age groups. These PVE’s were compared and averaged across participants and age groups, and mean Dice similarity values are shown by age difference from the participant. Note the consistent drop off in similarity, as utilized priors grow farther from the appropriate age range.

The “Image-AVG-*a posteriori*” method with age-appropriate MRI template was the best fit over both GM/WM and two IBSR groups. We therefore used the Image-AVG-*a posteriori* method to compare the age-appropriate segmentation as a proxy for the manual segmentation. The purpose of these ANOVA’s was to evaluate the effect of use of age-inappropriate priors, by examining the overlap between the segmented brain PVE’s based on the participant’s age-appropriate five-year template and successively older and younger five-year templates (see Figure 2). We conducted separate tests for GM and WM. For GM, a 14 (template age) × 26 (age difference) mixed ANOVA revealed a main effect of age difference, *F*_(25,492)_ = 17.68, *p* < 0.0001, and an interaction between age and age difference, *F*_(143,492)_ = 1.47, *p* = 0.0014, but no significant age main effect. The pattern of findings for WM were similar: a main effect of age difference, *F*_(25,492)_ = 7.76, *p* < 0.0001, and an interaction between age and age difference, *F*_(143,492)_ = 1.38, *p* = 0.0062. Figure 6 demonstrates the change in fit of successively older and younger five-year templates for four selected age groups (20–24, 40–44, 60–64, and 85–89). Figure 7 shows the changes for younger and older ages summed over all age groups. The same general pattern is present in all graphs. The overlap between segmentation based on age-appropriate and age-inappropriate templates decreases as the age is further from the participant’s age. We tested the age-difference main effect for all 14 age groups, and found a significant age-difference effect at each group. These *post hoc* tests shows that the interaction between age and age-difference occurred because of the differing patterns across different ages (e.g., Figure 6).

### Volumetric Analyses

**Figure 8:**
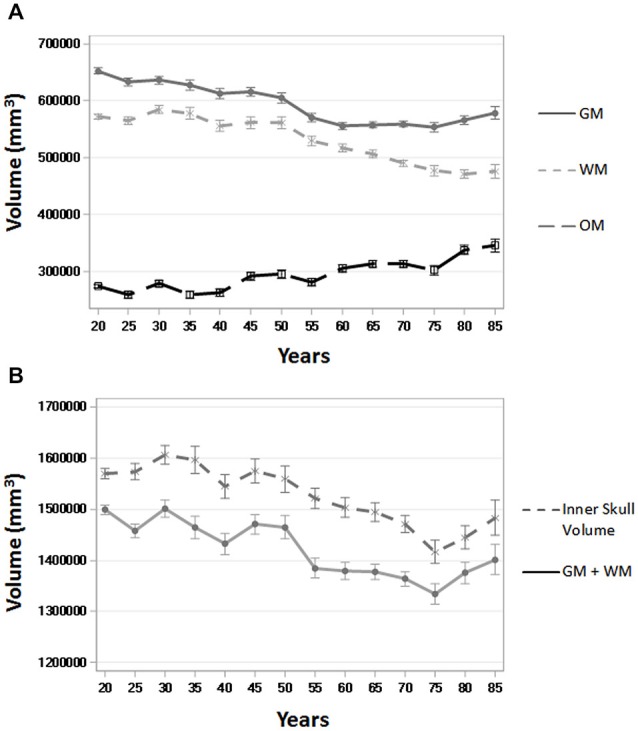
**Whole brain tissue and volume measures across age. (A)** Gray matter (GM), white matter (WM), and other matter (OM) volumes (in mm^3^) over five-year age group (all participants) using the PVE based on age-appropriate five-year template priors. **(B)** GM + WM and inner skull volume (in mm^3^) over five-year age group (all participants). Inner skull volume was taken from results of the betsurf program. Ticks on each bar represent +/− one standard error.

To assess the ability of age-appropriate templates to confirm previously discussed characteristics of brain aging, we calculated GM, WM, and OM volumes (in mm^3^) for all participants using the PVE volumes based on age-appropriate five-year template priors. The results closely match previous volumetric studies in that GM volume shows a linear decrease throughout the adult lifespan, whereas WM volume stays somewhat stable, even increasingly slightly up until age 50, and then starts to decline (Figure 8). OM volume shows a small, associated increase in volume, especially after age 50 (Figure 8). We also calculated GM + WM volume and as a point of comparison, we calculated inner skull volume (also in mm^3^) using results of the betsurf program (described in section 2.7). Results demonstrate that at younger ages, the volume of GM + WM closely approximates that of the inner skull volume but over age a separation occurs such GM + WM volume is not as large as inner skull volume (Figure 8). This separation is likely due to the loss in GM and WM volume accompanied by an increase in OM volume.

## Discussion

The purpose of the current work was to create a set of age-specific adult brain templates that could be used for anatomical image processing. Our rationale for creating age-specific templates as an alternative to study-specific templates or young-adult templates (e.g., MNI) was two-fold. First, we wanted to create age-appropriate templates that could be used for multiple studies and second, we wanted to create templates in increments small enough to meaningfully represent neuroanatomical changes over age. We were successful in creating a set of templates that meaningfully represent neuroanatomical changes that occur throughout adulthood. The templates are based on a large number of images (>1000) and a wide range of ages (20–89), and provide five-year, 10-year, and multi-year increments. The age-specific templates created for this study should provide increased accuracy when used for the tissue classification steps often used with structural MRI analysis pipelines. The templates used in the current study are available online.^4^

The issues with using brain templates derived from young adult data for studies with special populations have been well-documented (Huang et al., 2010). These limitations are especially apparent when using young adult reference data as a tool for segmenting images from a range of ages into the different tissue types. As a potential solution, studies investigating differences in brain volume with VBM began using study-specific templates to improve tissue classification results. Early investigations comparing a study-specific template to the MNI template for tissue segmentation found that the study-specific template provided less biased estimates (Ashburner and Friston, 2000; Good et al., 2001; Thompson et al., 2001). Similar to these studies, we found that age-specific tissue priors provided less biased tissue estimates than did MNI or other young-adult tissue priors, especially for older adults. Age-specific five-year or 10-year tissue priors performed similarly and may prove equally useful in practical application. Additionally, we found that age-specific tissue average priors created with MNI priors as a base reference, while better than simply using the MNI, provided more biased tissue estimates than age-specific tissue priors that did not start with MNI base priors, especially for older adults. Aging investigations using study-specific priors that start with MNI base priors may offer a large increase in accuracy over simply using the MNI (e.g., Lemaître et al., 2005), however our results suggest age-specific tissue priors that do not start with MNI priors as a base reference may provide even better results.

The database from which we constructed the templates had approximately equal numbers of males and females. Generally template construction used for young adults does not distinguish gender. Our templates come primarily from Caucasian participants, though the exact distribution of racial and ethnic groups is unknown. It has been shown by others that templates based on Asian young adult samples have markedly differing characteristics to those based on primarily Caucasian samples. For example, Lee et al. (2005) found that a standard Korean brain template created based on Korean adults was shorter, wider, and less in height compared to the ICBM-152 template. Tang et al. (2010) created a Chinese MRI brain template and found similar results. This suggests that age (Huang et al., 2010) and race/ethnicity (Lee et al., 2005; Tang et al., 2010) may both be important factors when considering MRI template usage.

We also evaluated the templates with a small external validation dataset not used in the creation of the templates. We did this by using the manually segmented images from the IBSR (Filipek et al., 1994; Rohlfing, 2012) and comparing the automated segmentation with our segmenting priors applied on those MRI volumes with the manually segmented GM and WM. The age-appropriate average priors created with no base reference (see Figure 2) were the best fitting method for the external validation data set. This method outperformed MNI priors, age-appropriate priors with the MNI as the base reference set, and young-adult template priors (see Figure 4). These results suggest our age-specific templates will provide more accurate tissue segmentation in future datasets. The IBSR data volumes might be considered a “gold standard” manual segmentation against which to compare the classification methods. These comparisons show how well the age-appropriate average priors performed relative to a manual segmentation.

The quality and usefulness of these age-specific templates is validated through their ability to replicate age-related volumetric change trends in the literature. We found changes in GM and WM over adulthood with the segmented PVE volumes based on the participants’ age-appropriate five-year template. Similar to prior studies, the results showed a linear decrease in GM volume over the course of adulthood (Good et al., 2001; Ge et al., 2002; Sato et al., 2003; Sullivan et al., 2004; Taki et al., 2004; Lemaître et al., 2005; Smith et al., 2007), a quadratic change in WM, with slight increases up to age 50, followed by decreases thereafter (Ge et al., 2002; Lemaître et al., 2005; Salat et al., 2009), and a slight associated increase in OM (Good et al., 2001; Lemaître et al., 2005; Smith et al., 2007). There was a small apparent increase in the amount of GM at our oldest two ages (Figure 8), which seems different than the regular decline in GM at these ages reported in other study. However, this may be due to sampling biases or other exclusion factors in these data. It is worth noting that a similar analysis of these adult data along with child and adolescent data from our “Neurodevelopmental MRI Database” (Richards and Xie, 2015) showed that the GM volume peak occurs somewhat earlier, in late childhood or early adolescence (e.g., 10–12 years). This implies that the age trends noted in several studies reflect only the changes from the youngest age of the participants in the study, generally young adults.

The method of template creation that we used here should prove useful to the research and clinical communities, in particular for researchers focused on neuroimaging and aging. We utilized publicly available image post-processing software programs (FSL, ANTS) to produce our age-specific templates. Similar to others (Fonov et al., 2011; Sanchez et al., 2012a,b), the age-specific templates were created through an iterative approach that minimized the influence of MNI *a priori* data and maximized the preservation of the different sizes, shapes and tissue distribution of the adult data. The processing pipeline refined the images recursively, such that the optimization procedure was applied to the data at different resolutions, with successively higher resolution during the nonlinear registration. Our pipeline procedure should be useful to others who wish to create adult templates based on different parameters using state-of-the-art averaging programs.

## Conclusions

Discrepancies between aging brains and the current reference data available continue to be an issue and the need for age-specific reference data is apparent. Several large-scale normative samples have provided the opportunity to address the need for age-specific brain templates. We implemented an automatic image-processing pipeline for template creation that has worked well with pediatric and infant images (Sanchez et al., 2012a,b) and created age-specific adult templates using state-of-the-art averaging techniques. The age-specific templates provide more precise *a priori* information to guide segmentation. Similar to study-specific templates (Ashburner and Friston, 2000; Good et al., 2001; Thompson et al., 2001), use of age-specific reference data will hopefully facilitate the generation of more reliable conclusions about the volumetric changes that occur with aging. The images from the current study complement our earlier studies and result in a database of normative age-appropriate average MRI templates across the lifespan (Richards and Xie, 2015).

## Conflict of Interest Statement

The authors declare that the research was conducted in the absence of any commercial or financial relationships that could be construed as a potential conflict of interest.

## Footnotes

^2^http://jerlab.psych.sc.edu/NeurodevelopmentalMRIDatabase/

^3^www.nitrc.org/projects/ibsr

^4^http://jerlab.psych.sc.edu/NeurodevelopmentalMRIDatabase/
